# Evaluation of introduction to UNU-Casemix grouper & IT in Casemix system workshop for health staff in Indonesia

**DOI:** 10.1186/1472-6963-12-S1-O5

**Published:** 2012-11-21

**Authors:** Azam Rahimi, Saperi B Sulong, Mohamed Amin Embi, Syed Mohamed Aljunid

**Affiliations:** 1 United Nations University International Institute for Global Health, Kuala Lumpur, Malaysia; 2 Department of Community Health, UKM Medical Center, Kuala Lumpur Malaysia; 3 International Training Centre for Case-Mix and Clinical Coding (ITCC), UKM Medical Centre, Kuala Lumpur, Malaysia; 4 Faculty of Education, Universiti Kebangsaan Malaysia, Bangi, Selangor, Malaysia

## Introduction

Implementation of Casemix system needs a well-organized and computerized system with well-trained and oriented staff, otherwise the system will fail. Improving knowledge and understanding of the funding system among staff and managers in hospitals and health systems can provide the groundwork for service improvements. The aim of this study was to evaluate the effectiveness of Introduction to UNU-Casemix Grouper & IT in Casemix System workshop which was conducted in Indonesia.

## Method

A quasi-experimental pre-test and post-test research design was conducted to evaluate the effectiveness of the program by measuring change in pre-test and post test data. Data was collected from health staff in Indonesia (representatives from selected Hospitals) who attended to case-mix workshop which was a five days course of classroom-based training conducted by United Nation University-International Institute for Global Health (UNU_IIGH). A 20-item questionnaire provided the data. The course aims to consolidate both the practical coding skills and theoretical knowledge through a number of assessment based exercises and interactive activities.

## Result

In total, 106 questionnaires were distributed and 91 (86%) were useable for the final analysis. The mean age of the participants was 37.85 years. 52.7% participants were male and 47.3% were female. 47.3% of the study sample had attended to similar workshop before. The participants’ mean score in the knowledge pre-test was 10.18, and 11.91 in post-test, which increased significantly (t=2.60, p =0.01).

## Conclusion

The finding of the evaluation of the workshop indicated that the program improved the participants' knowledge regarding case-mix system and highlights the role of education. It is unquestionable that education is a major factor in improving knowledge and understanding of the funding system among staff and managers in hospitals and health systems.

